# Visualizing aggregate movement in cities

**DOI:** 10.1098/rstb.2017.0236

**Published:** 2018-07-02

**Authors:** Michael Batty

**Affiliations:** Centre for Advanced Spatial Analysis (CASA), University College London, 90 Tottenham Court Road, London W1T 4TJ, UK

**Keywords:** movement, transport networks, flows, subway systems, cities and settlements, interactions

## Abstract

We argue here that despite the focus in cities on location and place, it is increasingly clear that a requisite understanding of how cities evolve and change depends on a thorough understanding of human movements at aggregate scales where we can observe emergent patterns in networks and flow systems. We argue that the location of activities must be understood as summations or syntheses of movements or flows, with a much clearer link between flows, activities and the networks that carry and support them. To this end, we introduce a generic class of models that enable aggregated flows of many different kinds of social and economic activity, ranging from the journey to work to email traffic, to be predicted using ideas from discrete choice theory in economics which has analogies to gravitation. We also argue that visualization is an essential construct in making sense of flows but that there are important limitations to illustrating pictorially systems with millions of component parts. To demonstrate these, we introduce a class of generic spatial interaction models and present two illustrations. Our first application is based on transit flows within the high-frequency city over very short time periods of minutes and hours for data from the London Underground. Our second application scales up these models from districts and cities to the nation, and we demonstrate how flows of people from home to work and vice versa define cities and related settlements at much coarser scales. We contrast this approach with more disaggregate, individual studies of flow systems in cities that we consider an essential complement to the ideas presented here.

This article is part of the theme issue ‘Interdisciplinary approaches for uncovering the impacts of architecture on collective behaviour’.

## 1. Introduction: defining movement

Patterns of human movement have been explored from classical times but it required a revolution in technologies which began with the industrial revolution to raise their prominence to systematic study. It was the internal combustion engine and mechanical vehicular technologies that developed in its wake that enabled cities to grow beyond the constraints imposed by how far we could walk which tended to limit the biggest cities to populations of less than a million. However as soon as the railway developed in the early nineteenth century, the ways in which people could move using such technologies became significant, not only because people could travel much further but because they could restructure their lifestyles in terms of where they lived and worked. One of the earliest descriptions of such patterns was made from a survey of all movements of traffic in the Pale of Dublin in the 1830s by the British Army. In 1837, Lieutenant Henry Harness produced a visualization of the flows within Pale which was the effective hinterland of Dublin [1] and his map provided a portent of things to come. The survey was specifically designed so that the British Government could figure out if there was enough traffic to build a railway, and these kinds of visualization are now used routinely to explore the impact of new transportation infrastructure. Many of the flow maps presented in this paper follow in Harness tradition including the famous map produced by Charles Minard in 1869 of the increasing and thence decreasing strength of Napoleon's army as it made its way to and from Moscow in 1812–1813 [2].

Human movements are initially recorded at the individual level and there are many plots of individual trajectories, as, for example, in very local contexts such as games where motion is key to the way the individual plays the game or the way a team confronts another on the field. The patterns produced by these kinds of situation often display aggregate structure and thus analysis has begun to explore aggregate trajectories and trails in search of a more generic spatial logic. In the case of cities, this is easy to see in terms of daily flows from suburbs to city and more locally with respect to retailing, which represent the predominant people and materials flows that are formed from the way contemporary cities have developed radially and concentrically around a central core. Our focus here is thus on spatial patterns that are aggregated from individual movements. Therefore, we will not present, explain or simulate the sorts of patterns and behaviours associated with fine spatial scales such as large-scale entertainment events where crowding is key. We will not discuss models of how individuals react to one another and their environment in generating emergent patterns, nor will we discuss the kinds of negative and positive feedbacks that determine how such patterns emerge. Our focus is thus on representing the patterns and visualizing their complexity in physical terms which can be clearly visualized in 2- (and 3-) dimensional Euclidean space, the key metric used by those disciplines and professions that aim to understand and then plan the built environment. We elaborate the wider implications for cities elsewhere [3].

There are many other perspectives on human movement that do not emphasize the kind of physicality that we assume here. Movement is intrinsic to the way people behave and even if they do not move physically, many social and economic relationships which tie individuals together imply communications that explain how individuals are positioned and located in space. For nearly 100 years, the social sciences have sought to explain power relations using networks while markets that define the way individuals and groups engage in exchange, trading with one another, imply the transfer of materials, money, and ideas. In fact, most social relations do invoke physical movement at some stage but in many discussions, this remains implicit. For example, the recent growth in network science has not focused very strongly on networks embedded in physical space for social networks which have driven applications are essentially topological and relational [4]. Here we will take the opposite approach rooting our presentation in explaining and visualizing patterns of movement that take place in physical space, often ignoring the detailed rationale for movement but always aware of the fact that our approach needs to be complemented by many other perspectives. In fact, the city is a many-facetted object or system of study and it is unlikely that there will ever be any comprehensive theory that relates all its dimensions. It is the example of a complex system *par excellence* [5] where a complete explanation of its form and function depends on many disciplines and scientific approaches.

The last feature of our study of movement which we need to emphasize relates to spatial scale. Although all the trajectories that we assemble here are traced from individual spatial behaviours, our two examples—from the multitude that we could envisage—aggregate these traces into patterns that are associated with intense dis-contiguous hubs in the city such as stations or districts where the populations are aggregated into at least the hundreds, sometimes the thousands. What we assume but do not have time to explain here is that if we were to disaggregate these to finer spatial scales, we would begin to see very different patterns for although there is a degree of self-similarity in cities as explored in the idea of the fractal city [6], explanations of urban phenomena, particularly movement patterns, differ at different scales. We do not have a good theory of how the many different kinds of patterns displayed at different scales can be integrated in a consistent set of explanations although we consider the purpose of this special issue of the journal is to assemble many different perspectives on such individual and collective phenomena; and we assume that this issue will enable readers to get some sense of the challenges in integrating different viewpoints and charting a way forward which enables us to integrate these ideas more effectively.

## 2. Locations and interactions

In this paper as we have already implied, we treat cities as large agglomerations of individuals who cluster together to pursue social and economic tasks to their mutual advantage. This advantage is defined as economies of scale which arise when individuals pool their labour and support their community with social relations. Cities are thus the hotspots in the economy, central to the way production and consumption are organized. Our usual model of their form is physical which we define at different spatial scales from the location of buildings at fine granularity through to entire metropolitan areas or regions whose morphology is much more coarsely configured. Since classical times, whenever there has been debate about cities, their representation has usually been in physical terms which we have come to call spatial where location and place are the key determinants of urban structure. Although cities evolve through time, most of our thinking about their form has been as if they are in equilibrium. They have been primarily explored at cross-sections in time while their dynamics has been, at best, implicit, largely because of severe limits on our ability to observe them in the aggregate or even at the level of individual behaviour over time.

Cities are thus primarily studied starting from their physical representation either at the scale of buildings which is essentially architectural or at the scale of the complete system, the entire town or metropolis which is essentially geographical. There is a strong disciplinary divide between these two perspectives which is reflected not only in what is articulated but also how their study takes place. At the finest scale, cities are essentially sets of buildings where the focus is on the building use, construction and design. In contrast at the city scale, the focus is on how different locations relate to one another in terms of their uses, their densities, and who and/or what occupies those locations. These may be anything from street addresses at the fine, small-scale to administrative units such as census tracts at the coarse, larger-scale. Thus the focus at the small-scale is essentially architectural and also possibly through the lens of engineering while at the large-scale, it is socio-economic. In this paper, we will select examples from each of these scales to give some sense of the main issues involved in questions of how their components—buildings to locations—relate to one another.

Here we will not be emphasizing the raw physicality of buildings and locations *per se*, for the study of cities is fast moving away from these perspectives to explaining how cities function in terms of patterns of movement. To this end, we need to begin to think about buildings and locations other than with respect to their autonomous representation but in terms of how they relate to one another. In this perspective, location no longer takes pride of place, it is the relationships between locations that are critical. In this sense, location can be seen as being a function of these relationships. The best example one might point to is residential housing. At any time of the day, the number of residents in any location will vary and this variation depends on the numbers who travel to or from the location in question for different purposes such as employment, education, retailing and so on. It is not really possible to explain the numbers at such a location without understanding these relationships that determine how many people reside, work, shop, go to school and so on in every other location. In this sense, then, we might think of locations as being functions of interactions or movement, of which there are many types, some spatial but some acting in non-spatial ways through electronic or social media. In any event, we treat these interactions as spatial aggregates where we see the clearest patterns emerging from individual movements associated with activities which ultimately are generated from the bottom up.

Although we have noted that cities tend to be studied and represented as though they are in equilibrium, movement in and of itself implies change and dynamics. Movements take place in time—they operate through time—but they also change on slower time scales just as the occupation of buildings and locations change with respect to their use and density. For the most part, here we will not study very long-term changes in movement but we will point to some of the research questions that need to be explored. Our problem in extracting the study of movement from the study of the city more generally is that our theories about cities are not well-formed and often confused—there is a science in the making but this is rudimentary [7]. We thus have to curtail our discussion of certain aspects of movement due to the fact that we are not able to discuss dynamics and equilibrium in great detail or questions about the evolution of cities and their complexity. Nevertheless, we will provide a rounded enough review to hopefully engage the reader in some of the key questions.

The current changes in our thinking about what is happening with respect to the study of location and movement in cities arises from many issues. First, it is logical to suppose that objects which are related to one another cannot be satisfactorily explained and understood without considering the set of relations within which those objects are embedded. Second, our current practice of design and planning has clearly demonstrated over the last century that simply assuming we can rebuild our cities without taking relationships between the objects that comprise them is wholly inadequate. We now know enough about cities to know that if we ignore how things are connected to one another, particularly with respect to their transport, all sorts of counterintuitive and undesirable effects can occur. The classic example is the problem of observing traffic congestion on a highway and then deciding to enlarge the road or build another in parallel to reduce the current flow volumes, only to find that both roads fill up with traffic as soon as the increased capacity becomes available. Interdependencies are everywhere in cities and we ignore them at our peril. Third, as we move headlong towards a digital society, many new patterns of movement through electronic transmission have come to dominate our cities. Email for example, now underpins most economic and a good deal of social activity, and social media is influential on what and where we do things in cities but so far it is largely a closed book with respect to the effects it is having on how are cities are organized. The substitution and addition of an online world alongside our material world is having enormous effects but we have little idea of what these are because we do not have good models to understand the importance of movement. All this raises important challenges that need to be resolved to enhance our understanding of how cities form and function.

There is one other important issue pertaining to a science of cities that we need to note before we focus on movement and this relates to measurement. In the past, most science has achieved its goals by defining systems as sets of objects that are subject to extensive and continually improving measurement. This has also been a precursor to good classification. When we enter the world of relationships between objects, it has been much harder to develop satisfactory measurements, largely because relationships are harder to define and harder to abstract. Relationships are less stable and even if they pertain to flows, these vary in time and hence are harder to identify. Capra & Luisi [8] have articulated this problem rather well and point to the difficulties of understanding relationships between objects in very clear terms when they say:

The shift of perspective from objects to relationships does not come easily, because it is something that goes counter to the traditional scientific enterprise in Western culture. In science, we have been told, things need to be measured and weighed. But relationships cannot be measured and weighed, relationships need to be mapped. (p. 80)

In this paper, we will take Capra & Luisi [8] at their word and demonstrate how movements need to be defined not only formally and through data but also in terms of their visualization. We will in fact map physical movements quite literally as cartographic patterns but also in terms of other ways of showing spatial relationships. Before we launch into models and methods for doing this however, we need to define how we might best represent movement in cities and to this end, we will define the wider domain in which such relationships exist.

## 3. Representing flows and networks

We will assume that cities can be represented as a set of locations whose attributes we will denote using indexes *i* and *j* where we assume that these pertain to locations that have an area such as a census tract or a point such as an address, geocode or geotag. These locate a point where a building is centred or the centroid of an area which contains some activity of relevance to the spatial system in question. We can identify at least two kinds of attribute that relate to movement: *F_ij_* which is the flow volume of activity or information between *i* and *j*; and *σ_ij_* which we define as the channel or network link between *i* and *j*. The network link might be measured as being present *σ_ij_* = 1 or not *σ_ij_* = 0 or it may have some attribute pertaining to its physical channel capacity etc. If we measure capacity as *q_ij_*, then it is easy to see how we might define the flow density as ∂*_ij_* = *F_ij_*/*q_ij_*.

These kinds of measurement are easy to make for material flows such as road traffic but for electronic they are much harder to observe. For social relationships as developed in studies of social power, neighbourhood association, and personal/cognitive interaction, they may well be almost impossible to define, notwithstanding that there is widespread agreement that they exist. Indeed, there are important contributions to understanding how cities work that do not refer explicitly to physical flows such as in the work of Lefebvre [9] among others and a rounded view of urban phenomena must embrace these related perspectives as we alluded to earlier. However, in measuring relationships where flows and networks are relevant together, then measures are often mixed, flows being based on people, or packets, or materials while networks pertain to the physical characteristics of channels. In one sense, flows pertain to activity locations while channels pertain to the physicality of the environment in the same way buildings are defined. This mixture can be confusing and one has to exercise great care in combining and comparing interactions which involve both flows and their networks as conceived in terms of channels.

We must first make two sets of distinctions between short and long time scales, and between fine and coarse spatial scales. Different kinds of movement take place across different types of space and over different time intervals, and this is further complicated by the fact that movements that occur frequently over short time periods might also change their form and function less frequently over longer time periods. The same is true for movements across spatial scales in that movements that take place, let us say within a building on a real-time basis, might change and can then be aggregated up to the neighbourhood scale. Many changes across these scales involve changes which show themselves in the individual elements at the finest scale. Again this can be potentially confusing because movement takes place in real time in any case and it is only when we aggregate it over space and time do we see different patterns across these scales. It is when these patterns change over longer time scales that we can better detect variations at more local spatial and temporal scales.

We need to make a simpler distinction with respect to time scales. In terms of time, we can define what we will call the ‘high-frequency city’ and the ‘low-frequency city’. The high frequency is the city that contains movements that occur in real time and can be observed in real time or near real time such as the movements of individuals or emails or energy flows that can be monitored and aggregated from seconds to minutes to hours and even to days, weeks and months. Beyond this, we are really dealing with the low-frequency city where months turn into years and where years add to decades, centuries, epochs, eras and so on. Typical differences might be between journeys made during the working day such as the journey to work from home compared to residential relocations that take place over years. To an extent all movement takes place in real time and the difference between the high- and low-frequency city is really one of how clear the patterns are at these different frequencies. The same kind of distinction takes place over spatial scales. At the finest scale, we are probably talking about people movements in terms of walking which define the scale at which these are recorded in contrast to transit movements that take place over wide areas such as the entire city region. In terms of temporal scales, movements are usually recorded second by second, or minute by minute or even hour by hour from real-time sensors but further aggregations tend to be generated from the finest real-time observations. When movements are recorded over months and years and decades, the actual movement is in real time but its aggregation is to much bigger temporal units. Spatial scale tends to be the focus of interest for all movement as it is recorded at the basic level although its aggregation to different spatial scales is usually based on what the focus of interest is, *high frequency, small scale* or *low frequency, large scale* which define the two key poles of interest in cities. We will use this simple classification to organize the presentation of our two demonstrations which follow.

In the last one hundred years, the main networks that have come to describe cities have been those based on transportation with road, rail, bus, walk and cycle modes being the most obvious and often sharing common physical infrastructure. Material flows which use these networks have also been separated from people flows but flows of information such as telegraph messages and telephone calls have barely been charted since their inception, notwithstanding early efforts to describe their significance to the form and function of the city [10,11]. Until the 1960s, computers were not generating flows of information in anything other than at the most local scale of the machines themselves and their off-line users but with the emergence of the Internet from that time on, email began to grow. With the development of the web, search, hand-held devices, and social media since the late 1990s, dramatic amounts of information are now circulating around cities which are probably having a major impact on many traditional patterns of location. All of these flows are pictured more with respect to their networks than the volume and capacities of their flow systems. To an extent, flows and networks are different sides of the same coin—one cannot exist without the other but it is the measurement of flows that is the most problematic, largely because of the invisibility of this data. Over longer periods of time, we can observe changes in where people live and work—changes which are implicit in migration patterns, and in measures of economic activity such as house prices and income and so on, all of which imply a degree of change, hence movement but in a non-spatial sense. The spatial dimension merges into the non-spatial when it comes to cities and this too reveals how complex the structure of the city is with respect to its dynamics and the way its economic markets interact with one another

In terms of data, physical networks are the easiest to observe with the growth in network science spurred on by the fact that many such networks are available for analysis. Flows are much harder to record. These have to be gathered using questionnaires which are expensive or by closed and robust automatic systems such as those used for recording transit payments. Many of the automated flow recording systems, beginning with analogue systems for recording flows of vehicles on roads, for example, cannot be integrated with data pertaining to those who generate these flows—the users—and hence the data, although accurate, is limited in its interpretability. Much data that would be useful such as electronic flow data e.g. email is largely invisible. It is so voluminous that even those who control the means of its distribution—the telecommunications companies—find it near impossible to make sense of the data for analyses that are important to their own study of movement for commercial purposes. Data on utilities is easier to measure but again invariably lacks any referent to use and where there is the prospect for such usage data as in the flow of electricity and related energy flows, making sense of these in socio-economic terms is limited.

Various models of flow and network systems have been proposed and we will note some of these below but much depends on the nature of the movement, the scale of resolution and the kind of data that is available from which a model might be estimated. Models of individual flows at the finest spatial and temporal scales have been proposed and many of the other papers in this special issue deal with such models. These are often referred to as agent-based in that each individual or object that is subject to movement is identified as a relatively autonomous agent and the simulation proceeds by modelling each agent's decision with respect to why and how they move. Often actual data for such models is sparse or lacking and thus many of these models tend to be exploratory and indicative rather than predictive. At the coarser scale, aggregations of individuals into populations is the focus of models at the metropolitan city scale and these tend to be less acquisitive of data and thus easier to estimate. We will introduce these below. Moreover, an increasing number of models which are taken from real-time data on movement are descriptive rather than predictive. The range of models and their mathematics is quite wide and we will selectively illustrate examples below to give some sense of the range of model types.

**Figure 1.**
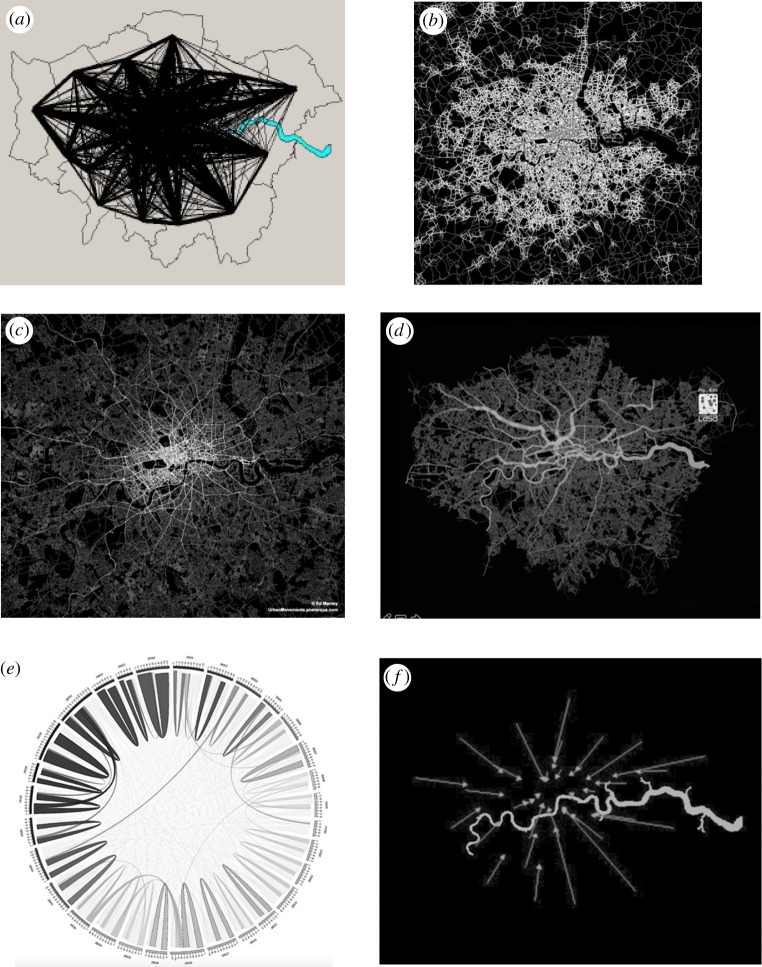
Different visualization of flow systems for Greater London. (*a*) Direct flows: desire lines; (*b*) London's road network; (*c*) flow assignment to the road network; (*d*) flow assignment to the subway network; (*e*) non-spatial direct flows; and (*f*) averaged vectors based on weighted averaging of direct flows. (Online version in colour.)

We also need to introduce methods for making sense of both movement data and models. This is increasingly the domain of visualization and as a prelude to this, we will illustrate some of these for patterns of movement in Greater London at relatively coarse spatial scales. Given a set of origins and destinations between which spatial movements take place, we can first visualize these as a set of flows that take no account of the physical networks on which such flows are based. These we show for the journey to work in figure 1*a* which is based on the coarsest aggregation into London boroughs which we call ‘zones’ while the underlying much more detailed road network is shown in figure 1*b*. If we then assign these flows to the network, we generate pictures of network flows as in figure 1*c* for the road system and in figure 1*d* for the Underground (subway) system. There are two problems with these visualizations and both relate to the level of detail needed. The self-flows, that is, the flows that remain within the zones which are called intra-zonal, are often much bigger than the inter-zonal flows that is *F_ii_* ≫ *F_ij_*, *i* ≠ *j* and these are visualized in the flow map in figure 1*e*. The other problem involves the level of detail of the spatial system in that as we increase the number of origins and destinations, the denser and more complex the data becomes and the more difficult it is to visualize. Figure 1*a* is a complete mess even for only 33 origins and destinations and therefore we need to simplify such flows. To resolve such problems of visualization, we need to move beyond a complete representation of each flow in map form and one way of doing this is to produce the vector fields that we show in figure 1*f*. These are weighted directional flows which are an average of all flows from particular origins to all destinations. This simply gives some of the tools that are necessary to make sense of movement data and to provide some idea of these challenges, we will now explore two examples in much more detail.

Last but not least, we need to make the point that in the models and data we are focusing upon, the behaviour is that of the individual not the physical system that this behaviour is contained within. Individuals which represent our basic objects or components that make up cities do not influence the configuration of space at least in terms of the high-frequency city. Over longer time spans, individuals may adapt their behaviour to the physical structure of the city or vice versa adapting the physical structure to their own behavioural needs but we will not deal with the latter models here. The models we will focus on enable us to predict movement largely at a cross-section in time—as if the city is in equilibrium even though we know it is not [12]—and we will emphasize how individual behaviours are aggregated to more macro types of behaviour in developing models at ever coarser spatial scales. Many of the models in the papers in this special issue deal with how the very local environment might be adapted by agents, particularly those that pertain to animal populations, but once we scale up to the city level, most individuals moving in cities at that level take their physical environment as fixed.

## 4. Movement in the high-frequency city

We begin with an object *k* which in our context is an individual or an aggregate of individuals engaging in movement for which we can define a probability 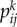 of that object or aggregate moving from one location to another. We refer to the first location as an origin *i* and the second as a destination *j* and we define the probability for that object moving as4.1
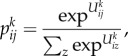
where 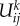 is the utility gained which is associated with the movement by *k* from *i* to *j*. The utility is usually defined as a weighted linear sum of benefits and costs associated with the spatial separation between and the activity located at the origins and destinations of the flow. Here we will specify this as a benefit 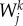 at location *j* and a cost of travelling 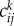 from *i* to *j*. We can aggregate across individuals or specify costs and benefits as aggregates of individuals although in terms of our first example involving movements at different times of the day, we will restrict our models to those simulating individuals. Using these definitions, our model thus becomes4.2
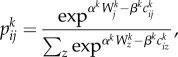
where the parameters *α^k^* and *β^k^* are determined so that the model fits observed behaviour in some best way. Finding a best fit for these models is an enormous subject area well beyond the scope of this paper just as the generalization of these models to wider developments in theories of choice opens the door to invoking ideas about individual perception of the utilities involved in undertaking movements of any kind [13,14]. In equation (4.1), the probability is defined with respect to the individual and thus it is a conditional probability of being located in *i* and moving to *j*. If this probability pertains to a larger group of individuals, then we can write the flows 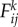 associated with the movement probability as4.3
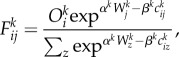
where 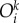 is the total number of individuals being aggregated into a class or group *k*. Note that this probability is normalized to this total if summed over *j*. If the utilities pertain to individuals rather than a group, then we can write the total flow for all groups as4.4
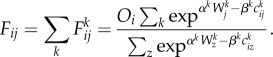


The summation over *j* gives the total of all individuals moving from origin *i* to all destinations *j* as *O_i_*.

There are many variants of these models that are adapted to different flow systems, but two classes stand out that are closely related. Their origins lie in analogies with gravitation and potential and were proposed as far back as the late seventeenth century just after Newton developed his basic mechanical equations. In the 1960s, these models were re-interpreted using ideas from statistical mechanics that provided a formal structure for their derivation as entropy-maximizing models [15]. They are still widely applied in transport modelling. In the 1970s, they were disaggregated and linked to individual-choice theory [13], and this provided a basis for much more detailed individual modelling [14]. These discrete choice models have been further developed to underpin a variety of micro-simulation models of transport activity such as the MATSim model [16], and as embedded in these kinds of agent-based activity frameworks, they now constitute the state of the art. Currently the limitations of these models with respect to the factors used by individuals to articulate travel costs are being addressed but progress is slow and faces the same kinds of problem which dominate choice theory in general.

We will sketch as our first example two applications of these kinds of models to individual travel behaviour. In essence, we assume that the benefits of making a trip from an origin to a destination depend on what is at the destination which we measure by 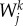 which for the journey to work would be employment or some variant thereof such as wages while the deterrent effect or disutility might be transport cost 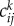. Note that the way these enter the utility function relates to their positive and negative effects on the amount of travel behaviour. In a system of origins and destinations, we can aggregate the individual flows to form the number of individuals at the origins that we assume we know and those that are predicted at the destinations. Now we need to build the model so that we can simulate the flows at different instants of time and in this sense, we have several variants of the basic model. We first annotate the flows by the time instant *t* as 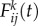 and we can specify utilities that vary with time too. Let us assume the most detailed model from equation (4.3) which can now be written as4.5
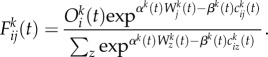
Using the basic model in equation (4.3) where we have 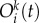 individuals of type *k* at the origin, then we have a total at each origin which is fixed as 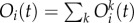 and an activity or population predicted at each destination 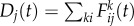. We can then compare these predictions at the destinations with those that we observe from data, having calibrated the model using an appropriate method as we implied above [14].

**Figure 2.**
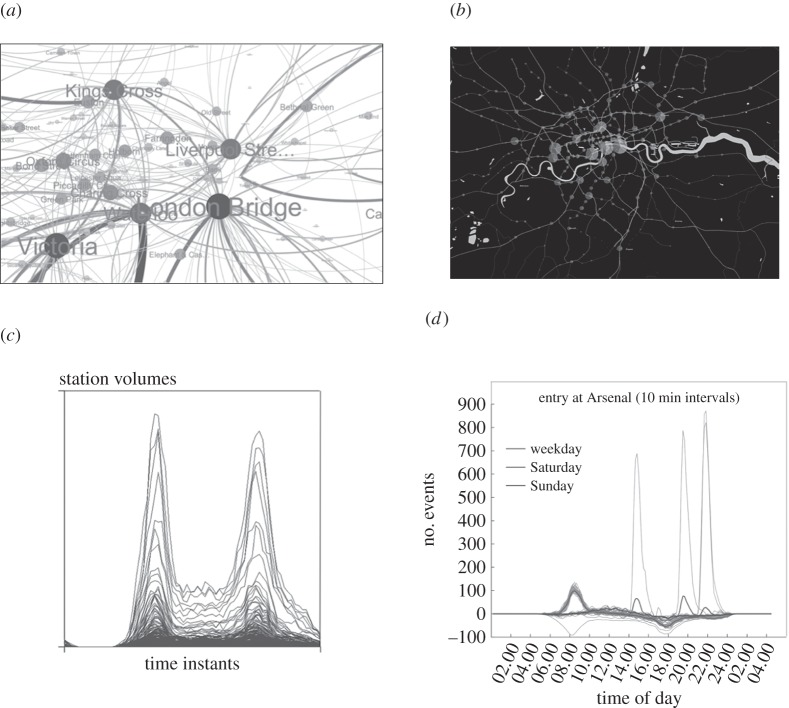
Travel volumes and profiles for the Underground (subway) in Greater London. (*a*) Abstracted flows within central and inner London; (*b*) exits and entries across the entire system; (*c*) daily trip volumes in all subway stations; and (*d*) morning, evening peaks and football matches at the Arsenal subway station during a synthetic week.

Our first application is to movements on the subway system in Greater London where we have excellent flow data from the Oyster card system which is used by 85% of all travellers using the network and where the order of magnitude of trips just on the subway made during a working weekday is around 6 million. Each movement is captured by the data and made available on a minute by minute basis which can be further aggregated into any appropriate but larger temporal unit. The data can be graphed in terms of desire lines between origins and destinations where each line pertains to the number of trips [17]. We show an example of this for a typical peak hour in figure 2*a*. Each origin is shown in terms of the total flow in this figure while in figure 2*b* we show the breakdown into origin and destination flows as proportional circles and their subdivision into these two types—entries and exits—for each hub that is a subway station. In figure 2*c*, we show the typical flow over time for the whole system. In figure 2*d*, we show a subway station (Arsenal, adjacent to the Emirates Stadium, where Arsenal FC play). The flows during the typical working weekday are dominated by morning and evening peaks but the extremely peaked flows are due to trip makers entering and leaving the station associated with football games. In this figure, we use a convention where we graph the exits from the subway station as positive net flows and the entries as negative.

We will not dwell on how we calibrate this model but there are many issues involving how the independent utility variables vary over the temporal intervals. Our goal here is simply to give the reader some sense of how we go about representing and modelling this kind of individual-based activity that can be easily aggregated to the system with which it is associated—a highly organized subway system with limited exit and entry points which has a very rigid physical configuration that forces behaviour to follow certain channels. Although there are many issues with this style of modelling, in this context it is the nature of the spatial system that has the most impact on the quality of these models. Such systems are highly constrained in that they are embedded within other transport systems that link different modes together. Many people travelling on transit systems such as the London Underground, also use less constrained transit and private transport systems as part of their overall patterns of movement. Trip makers must always walk some distance to gain access to a vehicular system and this kind of multimodal transport complicates the modelling process. It is possible to extend the models illustrated here to deal with more than one network and to enable networks to compete for the patronage of an individual traveller through different transport cost structures. One problem however is that data on multimodal trips is hard to assemble because different modes are captured in different ways. If the data is assembled by direct questionnaire, then there is some hope for comprehensive models but many new datasets such as the Oyster card system in London vary across different modes of transport. A traveller may use bus and subway and heavy rail to make a trip, and on each mode the use of the card differs, being comprehensive at both ends of the trip for subway, only being used to log the start or origin of a bus trip and varying dependent on the status of the users as to whether the card can or cannot be used on heavy rail. Walking between and to various transport modes depends on non-automated data which is hard to get although in time, some of this data might be acquired by automation using smart phone technology, subject of course to important limits on privacy and confidentiality. These then are some of the problems that plague these kinds of applications.

**Figure 3.**
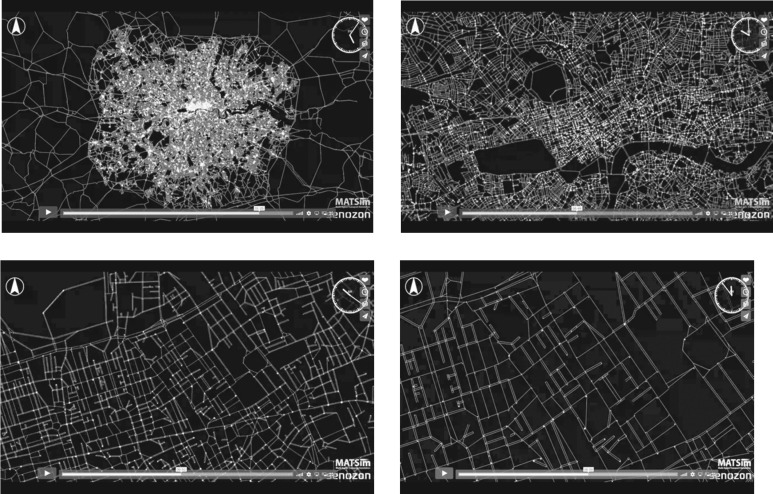
The London ABM MATSim model at different times and different scales. The dots represent moving travellers; for the animations see https://vimeo.com/119354430.

We noted above that the discrete choice models we have introduced here can be used to compute the probabilities of individuals making different kinds of trips during any period of time. These models treat individuals as agents and enable the sequence of origins and destinations that take place when individuals travel to be simulated. Essentially, each individual has a travel profile and time budget that needs to be met in terms of their daily activities. An individual will then generate trips to satisfy their daily activities schedule, and this leads to these trips being assigned to the network. Where they travel to is dependent on predictions from the models noted above, and these predictions are used to generate all the travel decisions during each individual's activity schedule. When all these trips are loaded (assigned) to the various networks, it is likely that the pattern is not feasible in certain ways and this leads to positive feedback that enables the individual traveller to make marginal changes in their schedules and locations that lead to another allocation. These changes hopefully lead to a convergence, hence a feasible pattern of trips which represent the solution. These models generate individual movements and hot spots of congestion while also generating trip volumes in aggregate at different locations as computed from models such as that in equation (4.5). We have built such a model for Greater London using highly disaggregate household data, which enables us to predict journeys to work over typical daily schedules [18]. An illustration of this kind of simulation is presented in figure 3 but to examine this in the requisite temporal detail, readers are directed to view the Vimeo movies: MATSim for London at https://vimeo.com/119354430, and TRANSIMS for Milton Keynes, UK at https://vimeo.com/33108792.

## 5. Movement in the low-frequency city

We now need to move to more aggregate spatial scales where we also deal with aggregated populations but before we do so, we need to note the generic nature of the models we are developing. The model types that we have introduced with their links to highly disaggregate agent-based simulations based on individual behaviour, or to social physics/spatial interaction models of aggregate populations, are of similar form and structure. However, when one disaggregates to really fine spatial scales such as the level of individual streets, these models become less appropriate, and models that rely less on purposive behaviour in the locational sense, such as those in space syntax [19], become more relevant. These models do not simulate trip makers being attracted to destinations that take travel cost into account but incorporate physical characteristics of crowds which avoid obstacles of various sorts producing flocking and related behaviours, while being grounded in cognitive perception. For example, many of the papers that deal with the movement of animal populations in this special issue deal with forces of a physical kind that determine how the objects of interest move and respond to their environment. They do not, however, deal with purposive behaviour of the human variety based on decision-making that attempts to optimize socio-economic costs and benefits, but, to a large extent, all these approaches are need to complement one another.

When we scale up to cities which we represent by subdivision into small zones or neighbourhoods, sometimes called TAZs (Traffic Assignment/Analysis Zones) which often have a few thousand trip makers located within, we usually adapt these to simulate all the trips that are generated in each of these zones. We can also use the same kinds of utilities based on benefits less costs that we specified in our generic equations listed previously in (4.1) to (4.5). In fact, the model we specified in these equations is what is called singly-constrained in that the flows or trips generated sum to the activity at the origins *i* while the model is designed to predict activity at the destinations *j*. Formally then 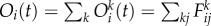 where we aggregate over *k* individuals and *j* destinations and 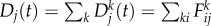 where we aggregate over *k* individuals and *i* origins. We still index these flows at a cross-section in time and insofar as there is any dynamics, it is able to enter these equations through the utility terms. But as such, there are no explicit dynamic processes based on feedbacks of the kind that are key to the way the city evolves. These models are still, at best, comparative static, meaning that future states based on changing the independent variables need to be compared with the existing state when this kind of ‘what if’ prediction is made with these models to inform the planning process.

We have built a variety of models based on equation (4.5) for different sectors in the UK space economy which we have defined from the population census geography called ‘middle layer super output areas’ (MSOAs). These zones contain on average around 7000 persons and there are 7201 in the model area which currently is England and Wales. The model is to be extended to Scotland shortly when the relevant data becomes available. What we show here is the journey to work model which links employment at origins *O_i_*(*t*) to destinations which enable us to predict the working population resident at those places as *D_j_*(*t*). We calibrate the model by simulating how close the predicted flows 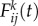 are to the observed 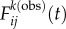 estimating the individual or group specific parameters *α^k^*(*t*) and *β^k^*(*t*) so that the average benefits and costs that the model reproduces match those of the data. The extended model simulates not only the journey to work but flows in the retail sector between population and commercial centres, as well as being extensible to include flows in the education and health sector which reflect journeys to school and to healthcare centres and hospitals.

**Figure 4.**
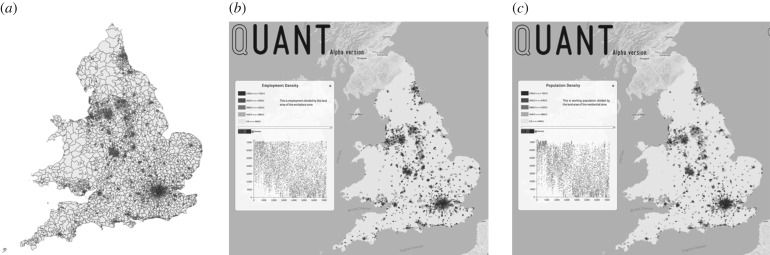
Scaling movement models up to the national level. (*a*) MSOA zoning system; (*b*) employment density; and (*c*) population density.

**Figure 5.**
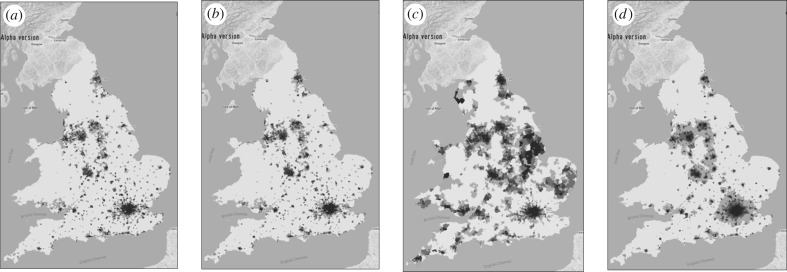
Observed and predicted population densities and accessibility. (*a*) Observed population density; (*b*) predicted population density; (*c*) differences in population density; and (*d*) population accessibility.

To illustrate the model, we show the zoning system for England and Wales in figure 4*a* and the distribution of employment and population in figure 4*b*,*c*. These distributions are quite similar and emphasize the fact that at this scale, we see the density of cities and related settlements which is a proxy for the density of movement. The model is web-based and can be run from any location (see http://www.quant.casa.ucl.ac.uk). In figure 5*a*, we show observed population again, in 5*b* predicted population, and in 5*c* the population differences as well as the observed accessibility to population from the employment sector as figure 5*d*. This measure of accessibility and there are many such measures which can be computed from these kinds of models, is based on potential values from the gravitational model [20], defined in this case as5.1
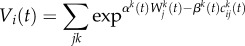

which is the competition or normalizing term from equation (4.5). *V_i_*(*t*) is a measure of nearness to residential population while population potential can also be computed in a symmetric way which gives a similar measure of nearness to employment. Accessibility measures are widely applied to look at the nearness or proximity of places to one another as summations of the influence of size and distance between a place to all others and in this sense, they represent a kind of simplified movement model where movements are collapsed to location.

**Figure 6.**
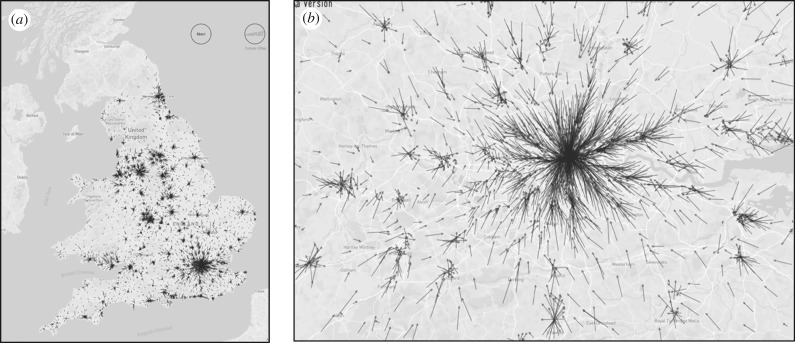
Average vectors computed from the journey to work flows between all 7201 origins and destinations in England and Wales. (*a*) Flow vectors in England and Wales; and (*b*) flow vectors in Greater London.

What is hard to visualize from these models are the flows or movements which give rise to far too messy and complex a pattern as we noted above but we can produce vectors or average flows as we did for Greater London in figure 1*f*. What we do is examine each flow 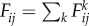 and consider this as a vector of length (*x_i_*, *y_i_*) → (*x_j_*, *y_j_*) of which there are 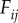. For each zone, we then add all these vectors to every other set of vectors and then take the average. This gives us the length and orientation of the average vector (Δ*x_z_*, Δ*y_z_*) centred on *i*. This is computed as5.2
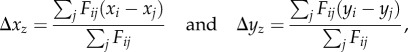
where the coordinates of the average vector from any point are given by (*x_i_*, *y_i_*) → (*x_i_* + Δ*x_z_*, *y_i_* + Δ*y_z_*). We show two examples of these flows for England and Wales and for Greater London in figure 6*a*,*b* and this gives a fairly clear picture of the orientation and strength of movements in these regions which accords to our common perception of the density and volume of these flows.

## 6. Next steps: challenges in simulating aggregate movement

As we have articulated movement here, we have assumed that dynamics in general relates to the time taken from when an individual or aggregate population starts its journey at one place and finishes at some other or the same location at a later time. The dynamics that is implicit in all our models is that movement over space takes time but that it is space that is more privileged in terms of the explanatory dimension, rather than time. This is all implicit in the high-frequency city and if we wish to explore how movements change over longer time frames, we must move to thinking about the low-frequency city and the forces that drive such change. As location is a function of movement, then explanations of longer term change—how the number of journeys change more slowly reflecting changes in not when to travel but where to travel on a semi-permanent basis—involve moving to models of location and this takes us way beyond the focus of this paper to questions about the evolution of cities [7].

There are, of course, other outstanding problems involving the kinds of movements we have described here. A key problem is how different kinds of movements dovetail and integrate with one another. It is difficult to track multimodal journeys because of limits on data—for example our Oyster card data for the London Underground is much richer than the same data for public bus because individuals only need to tap in on a bus whereas they tap in and out on the subway. This makes integrating rail and bus data problematic: independent travel data is required (if available) so that such stitching can take place, and this generates probabilistic outcomes rather than absolute predictions [21]. There are theoretical problems too in linking flow systems together. For example, many individuals engage in journeys to their work but at the same time are using email and social media to communicate essential features of their job to others while sometimes working from home, sometimes working on the road and so on. To get a good perspective on cities working as flow systems, we need much more powerful theory to enable us to make sense of all this complexity. This is far from forthcoming despite the fact that some progress is being made [22]. There are an increasing number of related datasets that might inform the kinds of movements that we have focused upon here, particularly those pertaining to mobile telephone calls. One of the best examples of such work is that generated at the Senseable Cities Lab where a variety of telecoms data has been simulated and visualized to show key hotspots in cities [23] but this is a very active focus in research on the kinds of patterns explored in this paper, and substantial progress for enriching our understanding of mobility in cities is likely to come from such developments in the next decade [24].

The link between flows and networks is still problematic, particularly in the examples shown in this paper that represent flows as desires on the part of a population. These flows tend to be quasi-independent of the network system itself. In short, network science has tended to proceed quite independently of models of flow systems. Moreover, the relationships between the physical aspects of networks and the behavioural requirements and motivations of those travelling are not well worked out. Individuals moving, say, from the suburbs to the central city have many possible routes to choose from and may choose those based on the interaction of somewhat idiosyncratic factors in comparison with the more straightforward demands of getting from one location to another. This intersection of the physical with the social and economic is an age-old problem in thinking about cities but in many respects, it is likely to be somewhat more tractable from others we have raised. In progressing these issues, visualization is ever more important, and considerable progress needs to be made in visualizing such patterns in space–time as well as in broaching the question of how different spatial and temporal scales can be simultaneously visualized to provide a much more integrated understanding of movement patterns in cities [25]. These then represent directions for future work, and the interdisciplinary focus of the papers in this special issue help in their definition.

## Data accessibility

This article has no additional data.
